# From Vineyard Soil to Wine Fermentation: Microbiome Approximations to Explain the “*terroir”* Concept

**DOI:** 10.3389/fmicb.2017.00821

**Published:** 2017-05-08

**Authors:** Ignacio Belda, Iratxe Zarraonaindia, Matthew Perisin, Antonio Palacios, Alberto Acedo

**Affiliations:** ^1^Biome Makers Inc., San FranciscoCA, USA; ^2^Department of Microbiology, Biology Faculty, Complutense University of MadridMadrid, Spain; ^3^Department of Genetics, Physical Anthropology and Animal Physiology, University of the Basque CountryLeioa, Spain; ^4^IKERBASQUE – Basque Foundation for ScienceBilbao, Spain; ^5^Laboratorios Excell IbericaLogroño, Spain

**Keywords:** NGS, wine microbiome, vine health, soil microbiome, metagenomic analysis, bioinformatic tools and databases, 16S rRNA gene sequencing

## Abstract

Wine originally emerged as a serendipitous mix of chemistry and biology, where microorganisms played a decisive role. From these ancient fermentations to the current monitored industrial processes, winegrowers and winemakers have been continuously changing their practices according to scientific knowledge and advances. A new enology direction is emerging and aiming to blend the complexity of spontaneous fermentations with industrial safety of monitored fermentations. In this context, wines with distinctive autochthonous peculiarities have a great acceptance among consumers, causing important economic returns. The concept of *terroir*, far from being a rural term, conceals a wide range of analytical parameters that are the basis of the knowledge-based enology trend. In this sense, the biological aspect of soils has been underestimated for years, when actually it contains a great microbial diversity. This soil-associated microbiota has been described as determinant, not only for the chemistry and nutritional properties of soils, but also for health, yield, and quality of the grapevine. Additionally, recent works describe the soil microbiome as the reservoir of the grapevine associated microbiota, and as a contributor to the final sensory properties of wines. To understand the crucial roles of microorganisms on the entire wine making process, we must understand their ecological niches, population dynamics, and relationships between ‘microbiome- vine health’ and ‘microbiome-wine metabolome.’ These are critical steps for designing precision enology practices. For that purpose, current metagenomic techniques are expanding from laboratories, to the food industry. This review focuses on the current knowledge about vine and wine microbiomes, with emphasis on their biological roles and the technical basis of next-generation sequencing pipelines. An overview of molecular and informatics tools is included and new directions are proposed, highlighting the importance of –omics technologies in wine research and industry.

## Introduction

Wine is a product with high sociocultural interest. In particular, wines with distinctive autochthonous properties have a great demand among consumers and collectors, causing important economic consequences. It is well known that physical (climate) and biological factors (soil, grape variety and fauna), as well as viticulture and enological techniques work together to determine the sensory-characteristics of a wine from a particular region, establishing the concept of *terroir*. In this sense it should be noted that, apart from these factors, recent studies highlight the contribution of the native vine microbiota in the winemaking process of wines from a particular region (Knight et al., 2015; Bokulich et al., 2016). Additionally, results from Burns et al. (2016), Grangeteau et al. (2017) correlate human-agronomical practices in vineyards with the soil and grape microbiota and, also with its later behavior at cellar, reinforcing the interdependence between the anthropogenic and microbiological basis of *terroir*.

Microbes transform plant products into socio-economically important products and fermented beverages, such as wine, which is an extremely important sector for several countries. For instance, the International Organization of Wine and Vine (OIV) estimated in 2015 that the global wine-growing surface area was 7,534,000 hectares, with the biggest producer being Italy (18% of the global total), followed by France (17.3%) and Spain (13.5%). Outside the EU, the USA has the highest wine production followed by Argentina, Chile and Australia (OIV, 2015).

Due to the economic importance of the grapevine, this crop has received considerable interest among researchers; although this attention mainly focuses on the plant genome and transcriptome/metabolome to better understand how the plant responds to the physical environment, abiotic stresses and diseases (e.g., the International Grape Genome Program, IGGP). However, plants cannot be considered a self-contained, isolated organism, as plant fitness is a consequence of the plant *per se* and its associated microbiota (Vandenkoornhuyse et al., 2015). Thus, a more holistic conception should include plant-microorganisms and microbe-microbe interactions.

Although the role of microorganisms at cellar stages has been well investigated, the biological aspect of soils has not received similar attention, when actually it contains a great microbial diversity with important roles in plant nutrition and health (Compant et al., 2010; Bhattacharyya and Jha, 2012). Next-generation sequencing (NGS) approaches have uncovered a higher than expected microbial diversity in both vine and wine and discovering new microbial species, some with unknown contributions to the organoleptic properties of wines (Bokulich et al., 2016). Stable differences among microbial populations of grape musts have been attributed to grape variety, geographical area, climatic factors and vine and grape health, leading to the concept of vine microbial *terroir* (Bokulich et al., 2014). This fact has been reinforced at a phenotype-metabolome level by other works such as Knight et al. (2015), Bokulich et al. (2016), and Belda et al. (2016). The later observed distinctive and clustered metabolic profiles (production of hydrolytic enzymes) for yeast strains depending on their geographical origin. It has been also observed that the origin of these microorganisms in musts is the microbial consortia of grapes, with the original reservoir of these microorganisms being vineyard soil (Zarraonaindia et al., 2015). Thus, the microbiological aspects of wine production are influenced by the vineyard and not just by the winery and fermentative processes.

The maturation of grapes is a complex process that depends on numerous factors (Kennedy, 2002). Traditionally, the most common measured parameters include: sugar concentration, acidity and aromatic and phenolic maturity. However, soil and grape microbiological complexity throughout the cycle of the vine and grape maturation is rarely taken into consideration.

**FIGURE 1:**
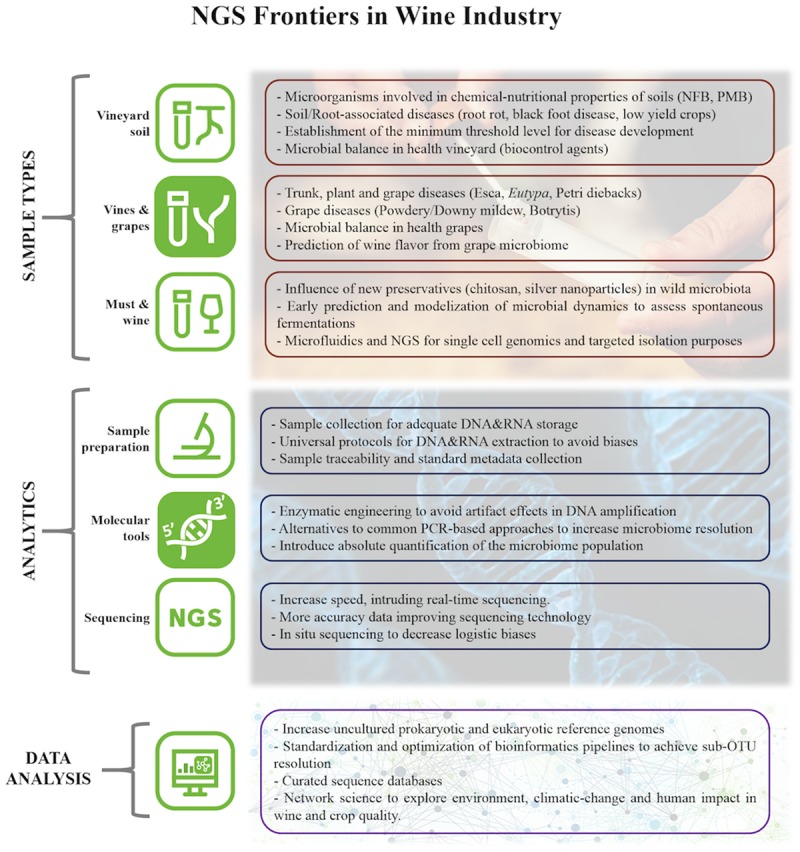
**Current challenges on viticulture and enology assumable by NGS approaches; advisable technical improvements; necessities and perspectives in data science.** NFB means ‘Nitrogen Fixing Bacteria’ and PMB means ‘Phosphate Mobilizing Bacteria’.

Communities of microorganisms (fungi, yeast and bacteria) associated with the vineyard play an important role in soil productivity as well as disease resistance developed by the vine. It is important to understand the microbial consortia associated with particular diseases, such as *Esca*, *Eutypa*, *Botryosphaeria*, and *Phomopsis* diebacks, and also the dynamics of infection processes in order to take preventive actions, especially at the most critical moments (**Figure 1**). For instance, microbial insights are crucial for defining strategies for the preparation of new plantings. At this stage, it could be interesting to improve the microbiological conditions of the soil by bioremediation and to avoid risk of cross infection during pruning (Bertsch et al., 2013; Fontaine et al., 2016).

The diversity and number of microorganisms that are able to establish in an ecological niche in the soil and on the vine will determine both the grapes’ health and the variability of microorganisms that will be introduced in the winery that further affect the fermentation processes and wine maturation (Barata et al., 2012). Thus, with adequately managed microbiome information, it could be possible to prevent fermentation problems, volatile acidity increases, *Brettanomyces* contamination and biogenic amines production. Knowing more about the microbiological conditions of the vineyard allows the winegrower to think about the reduction of chemical treatments and performing them only when they are objectively necessary. Additionally, this knowledge would help the winemaker to use lower sulfur concentration at cellar stages and even to decide the type of yeast and dose to be inoculated if and when necessary (**Figure 1**). This is valued information especially considering new enology trends, such as organic wines.

Next-generation sequencing technologies enable the detection and quantification of microorganisms present in vineyard soil, grapes, as well as its transformation later in winery. The impact of the microbiological component of *terroir* and how it contributes not only to its quality but also in the organoleptic features of the wine is considerable. This impact also contributes to the sensory regional distinctiveness and the wine style of the winery that currently plays an important role in differentiation and competitiveness in the worldwide market. If something can distinguish one vineyard from another, among other factors, it certainly is its microbial community. In this context, the objective of this review is to summarize the current knowledge about the role of microbial communities in viniculture, highlighting the contributions of NGS technologies and identifying new scientific-industrial frontiers.

## The Microbiome of Vine and wine: A Review

Plants host a variety of microorganisms (fungi, yeast, and bacteria) on and inside organs and their surrounding soil. Among these inhabitants are both harmful and beneficial microbes that are involved in crucial functions such as plant nutrition and plant resistance to biotic and abiotic stresses, hence in plant growth promotion, fruit yield, disease resistance and survival (Lugtenberg and Kamilova, 2009; Compant et al., 2010; Bhattacharyya and Jha, 2012).

Studies on microorganisms associated with grapevines have been centered on the cultivable fungi (mainly yeast) or bacteria that can have a negative economic impact, compromising the yield and quality of the grapevine, as well as wine production. Studies have focused on disease causing pathogens (*Agrobacterium vitis, Xylella fastidiosa, Erysiphe necator, Phomopsis viticola, Fusarium* spp., etc.) and microorganisms of enological interest. The later species have been grouped into three classes [reviewed in Barata et al. (2012)]: (1) easily controllable or innocent species, without the ability to spoil wine when good manufacturing practices are applied; (2) fermenting species responsible for sugar and malic acid conversion; and (3) spoilage *sensu stricto* species responsible for wine alteration The most widely known cultivable bacteria are acetic acid bacteria (AAB; e.g., *Acetobacter and Gluconacetobacter*) and lactic acid bacteria (LAB; e.g.*, Lactobacillus, Oenococcus*, and *Pediococcus*). Among yeasts, *Saccharomyces* members have attracted most of the attention as they are the main fermentation agents commonly used as inocula (e.g., *Saccharomyces cerevisiae, S. bayanus, S. pastorianus*, and *S. paradoxus* among others), while other genera are the most frequent wine spoilers (e.g., *Brettanomyces/Dekkera, Issatchenkia, Zygoascus*, and *Zygosaccharomyces*).

While culture dependent methods have been useful to detect and identify microbial organisms associated with grapevine and grape products, and also to study *in vitro* their metabolic properties (Belda et al., 2016), they have led to a rather biased picture of the microbial community. These methods neglect the larger, non-culturable fraction that is believed to be as high as the 95–99% of the microorganisms present (Amann et al., 1995; Curtis, 2002). In wine environment, due to the stressful environment associated to the addition of SO2, high ethanol concentration, etc., a fraction of the bacteria and yeast enter in a Viable But Non-Culturable state (VBNC) (Millet and Lonvaud-Funel, 2000; Divol and Lonvaud-Funel, 2005). At this state cells do not grow on culture media, however, they are still viable and maintain a detectable metabolic activity (Yamamoto, 2000) which may affect fermentation performance as well as flavor. Examples of such microorganisms include *Candida stellata, Brettanomyces bruxellensis, S. cerevisiae, Zygosaccharomyces bailii*, etc. (Salma et al., 2013). Thus, in order to reach to these VBNC microbiologists were driven to develop alternative culture-independent techniques. Particularly, quantitative real time PCR (qPCR) has been widely used to detect bacteria and yeast considered to be wine spoilers and that have VBNC strains responsible for the production of off-flavors or having a negative impact on wine, e.g., *Brettanomyces* spp. (Tofalo et al., 2012). Nowadays, qPCR is believed to be a rapid diagnostic tool to detect the presence and quantify the abundance of particular microorganisms of interest, however, when the objective is not a targeted species, but rather a whole community analysis, PCR-DGGE has been the classical method of choice. The later technique is adequate to approximate the total community profile and for comparative community structure analysis, but it has several drawbacks mainly associated to biases related with species richness estimates and its low sensitivity to detect low abundance species (Neilson et al., 2013). For instance, multiple bands could associate with single isolates. In addition, multiple sequences might be associated with a single band and preferential amplification biases between phylogenetically diverse members of the community have been shown (Neilson et al., 2013). Andorrà et al. (2010) compared the population dynamics of microorganisms of grape must fermentation by three culture independent techniques (DGGE, direct cloning of amplified DNA, and qPCR) with plate counting, and evidenced that the biodiversity observed in the must and at the beginning of fermentation was much higher when DGGE or direct cloning were used. However, the predominance of certain yeast such as *C. zemplinina* and *S. cerevisiae* during fermentation limited the detection of low abundant species. Thus, while DGGE is believed to give a quick and non-expensive view of the community, it skews microbial diversity estimates (David et al., 2014) and it has a limited use to study diverse environmental samples dominated by few species (Andorrà et al., 2010). When adding NGS technique into the detectability comparition of culture independent techniques to study yeast community in must and ferments, the studies evidenced that larger numbers of yeast species were detectable by NGS than by PCR-ITS-RFLP or DGGE in grape samples. Moreover NGS detected species in ferment samples that were undetectable with the two later techniques (David et al., 2014). In addition, Wang et al. (2015) analyzed Carignan and Granache grape must and fermentation from three vineyards in Priorat (Spain) and found that NGS detected all the species identified by the rest of methods (DGGE, qPCR and culture dependent), whereas DGGE could just detect the dominant species of non-*Saccharomycetes* class. Thus, NGS showed to be more appropriate to understand must and wine environment yeast communities (David et al., 2014; Wang et al., 2015).

Next-generation sequencing technologies are providing a powerful approach to achieve a more complete understanding of the complexities of microbial communities and their impact on plant growth, disease resistance/susceptibility, climate adaptation and environmental remediation. This technology is enabling researchers to simultaneously obtain information on thousands of taxa as opposed to targeted approaches that detect only a taxonomically predefined group. Thus, metagenomics coupled with new bioinformatics tools, is allowing performance of more complex multifactorial analyses and is becoming a powerful strategy in diagnostics, monitoring, and traceability of products. Its application in viticulture while recent is promising (**Table 1**), as accumulating data suggest that there is a much higher microbial diversity associated both with the plant (Leveau and Tech, 2010; Pinto et al., 2014; Zarraonaindia et al., 2015) and the fermentation process (Bokulich et al., 2012; Piao et al., 2015; Pinto et al., 2015; Portillo and Mas, 2016; Stefanini et al., 2016) compared to previous culture based studies. Most metagenomics research in this field has focused on microbial monitoring during fermentation to obtain a detailed description of the relevant microbial populations associated with grape and must that might lead to wine spoilage, an advance highly valuable for winemaking. These NGS-enabled studies reflect a wider range of bacteria, besides the commonly detected LAB and acetic acid species, able to persist in fermenting musts of various grape varieties (Bokulich et al., 2012; Piao et al., 2015; Portillo and Mas, 2016; Stefanini et al., 2016). For instance, the first wine-related study conducted in the wine environment with NGS was conducted by Bokulich et al. (2012) during botrytized wine fermentation using 16S rRNA gene amplicon sequencing. These authors showed an array of fluctuating low abundant taxa not traditionally associated with wine, as well as atypical LAB communities during the process. Similarly, results from Portillo and Mas (2016) suggested that AAB are more abundant and dynamic than previously thought during low or unsulfited wine fermentations, and seemed to be independent of the grape variety. Interestingly, in this study yeast diversity and dynamics during wine fermentation was assessed in addition to bacteria, evidencing that the genera *Hanseniaspora* and *Candida* were dominant during the initial and mid- spontaneous fermentation of Grenache grapes while certain *Candida* and *Saccharomyces* species predominated at the end of the fermentation. Other studies have demonstrated how different fermentation techniques (spontaneous *vs.* inoculated) affect the microbial community composition and its succession during fermentation (Piao et al., 2015), and also how the previous agronomical practices in the vineyard could play a critical role in these population dynamics (Grangeteau et al., 2017). These authors’ results indicated certain phyla are associated with each particular technique. Interestingly, they observed that *Gluconobacter* experienced a notable increase during organic fermentation, which led the authors to conclude that this might explain the increased susceptibility to wine spoilage in wines produced using that technique.

**Table 1:** Research and industrial hallmarks of viticulture and enology led by NGS approaches.

Enological features addressed by microbiome study	Reference
*Interference of microorganisms in plant physiology*	Lugtenberg and Kamilova, 2009; Compant et al., 2010; Bhattacharyya and Jha, 2012; Martins et al., 2013; Vandenkoornhuyse et al., 2015
*Microbial diversity in vineyard*	Leveau and Tech, 2010; Pinto et al., 2014; Zarraonaindia et al., 2015
*Microbial diversity in wine fermentations*	Bokulich et al., 2012; Piao et al., 2015; Pinto et al., 2015; Portillo and Mas, 2016; Stefanini et al., 2016
*Anthropogenic-agronomical practices determining vineyard and wine microbiota*	Burns et al., 2016; Grangeteau et al., 2017
*Microbial contribution to wine chemistry*	Verginer et al., 2010; Bokulich et al., 2016
*Terroir markers (microbial zoning)*	Bokulich et al., 2014, 2016; Burns et al., 2015; Knight et al., 2015


These above-mentioned studies enhance our understanding of microbial diversity during fermentation and allow the identification microbial contamination sources. However, as DNA sequencing approaches detects living as well as dead microorganisms, it is still not clear to what extent these microorganisms metabolically are active and capable of affecting organoleptic properties of wine. The role of the microbiota influencing the flavor, color and quality of wine, under a systems biology perspective, remained elusive until recently.

While soil, weather, farming techniques and grape variety contribute to the unique qualities of wine, adding distinctiveness and thus market value, the contribution of the microbiota in defining *terroir* is now in the spotlight of scientific research. Regionally distinct wines are highly appreciated by consumers and add value to the industry. In Spain alone there are 90 zones, which produce distinct so-called PDO wines, of which 69 are Denomination of Origin (DO), 2 are Qualified Denomination of Origin (DOCa), 7 are Quality Wine with a Geographical Indication (Vino de Calidad) and 14 are Single Estate Wine (Vino de Pago). While the chemosensory distinction of wines from different growing regions has been previously established [e.g., Loópez-Rituerto et al. (2012)], indigenous microorganisms associated with grapes were shown to be able to produce compounds responsible for the regional flavors of the resulting wine, e.g., VOCs (Verginer et al., 2010). In addition, Knight et al. (2015) experimentally demonstrated that wine organoleptic characteristics are affected by the origin and genetics of wild *S. cerevisiae* natural strains, providing objective evidence for a microbial aspect to *terroir*. Bokulich et al. (2014) showed that Cabernet Sauvignon must from different growing regions in California could be distinguished based on the abundance of several key fungal and bacterial taxa. This differential must microbiota could potentially influence wine properties and contribute to the regionalization of wine. The later was further proved in Bokulich et al. (2016); these authors demonstrated that both grape microbiota and wine metabolite profiles were able to distinguish viticultural area designations and individual vineyards within Napa and Sonoma Counties in CA, USA. Interestingly, the vineyard microbiota correlated with the chemical composition of the finished wines, hinting at the possibility of predicting wine phenotypes prior to fermentation. Nevertheless, wine aroma is defined by hundreds of chemical compounds with different natures (i.e., higher alcohols, esters, fatty acids, terpenes, thiols) causing a broad spectrum of sensory thresholds, and also suffering synergies and antagonisms (Belda et al., 2017). Thus, looking for microbial signatures determining wine typicity, the sensorial characterization of wines should consider not only chromatographic analysis (revealing the diversity and concentration of aroma compounds), but also developing serious sensorial or olfactometry analysis to reflect the real perception of wine aroma or, at least, considering odor activity values (OAVs) to correlate the real influence of microbial species in wine aroma, as was addressed by Knight et al. (2015).

While grape and must have been more heavily researched, Zarraonaindia et al. (2015) further hypothesized that the soil and its associated microbiota influences wine characteristics. First, per these authors’ studies, the aboveground bacterial community was significantly influenced by soil edaphic factors such as total carbon, moisture and soil temperature, which would ultimately impact the quality of grapes due to changes in nutrient availability for the plant. Second, soil bacterial communities differed between the sampled vineyards in Long Island, New York and those differences were reflected in the microbial composition in vine roots. These root endophytes can shape the microbial assemblages of aboveground organs by changing the endophytic microbial loads in grapes. Third, a significant input of soil microorganisms to grapes through epiphytic migration during harvest was suggested. The later was also evidenced by Martins et al. (2013), leading Zarraonaindia et al. (2015) to propose that soil derived microorganisms could have a greater role than previously anticipated in wine, as they will ultimately end up in the fermentation tanks. The link between soil microbiota and *terroir* was further evidenced by Burns et al. (2015) who identified distinctive microbial community profiles by American Viticultural Areas (AVA).

## NGS Microbial Profiling: Key Steps, Biases and Limitations

The above summarized studies were conducted on grapevines and wine and address microbial composition by means of 16s rDNA PCR amplicon and ITS (Internal Transcribed spacer) NGS sequencing for elucidating the bacterial and fungi community, respectively. This marker gene amplification and sequencing method, also called amplicon sequencing, has become the method of choice to simultaneously detect multiple species in must and wine environment since 2012 (see Bokulich et al., 2012, 2016; Pinto et al., 2014, 2015; Knight et al., 2015, among others). However, the particular experimental question of the research to be conducted will determine the method mostly suited to answer to the question. For instance, if the goal is to track a particular microbial strain or genus from soil to must to fermentation, then qPCR could be a more appropriate and has the added benefit of absolute quantification (Neeley et al., 2005). To detect a specific microbe, primers must be designed to be highly specific for the microbe of interest. Often the primer design can be completed by genome comparison of targeted and non-targeted strains to find a unique gene or region. Another strategy involves targeting a conserved gene (16S rRNA, *gyrB*, *rpoB*) and making sure the primers mismatch off-target strains particularly at the 3′ end. Single copy genes provide an added bonus for absolute quantification. Microbe quantification by qPCR, however, does not scale easily if the goal is to analyze more than a few strains while amplicon sequencing is suited to determine the community.

However, amplicon sequencing is not free of pitfalls, and different biases have been described in multiple steps of the process; First, DNA extraction method is one of the key and limiting steps for metagenomic analysis by NGS. Various approaches have been applied for environmental DNA extraction, including freeze–thaw lysis (Herrick et al., 1993), bead beating (Miller et al., 1999; Courtois et al., 2001; Urakawa et al., 2010; Petric et al., 2011), liquid nitrogen grinding (Ranjard et al., 1998), ultrasonication (Picard et al., 1992), hot detergent treatment (Holben, 1994), use of strong chaotropic agents like guanidinium salts (Porteous et al., 1997), and high concentration of lysozyme treatment (Hilger and Myrold, 1991). Furthermore, soil, grapes and wine are complex physicochemical environmental samples that contain many interfering agents for molecular analysis such as impurities, phenols, humic acid, fulvic acid, metal ions and salts, and therefore additional purification steps are necessary which can introduce bias by altering the original community (e.g., a fraction of the community might be lost through purification, etc.). There are several commercial kits that could be used to fasten the process, however, the selection of the best DNA extraction method and kit is not straightforward as different DNA extraction methods can produce different results (Keisam et al., 2016). Unfortunately, there is no “gold standard” for DNA extraction method and one should be selected on a case-by-case basis considering the aims, specimens of the study and scalability (including simplicity, cost effectiveness, and short handling time) and intended study comparisons. An additional problem is the introduction of contaminating microbial DNA during sample preparation. Possible sources of DNA contamination include molecular biology grade water, PCR reagents and DNA extraction kits themselves. Contaminating sequences matching water-and soil-associated bacterial genera including *Acinetobacter, Alcaligenes, Bacillus, Bradyrhizobium, Herbaspirillum, Legionella, Leifsonia, Mesorhizobium, Methylobacterium, Microbacterium, Novosphingobium, Pseudomonas, Ralstonia, Sphingomonas, Stenotrophomonas*, and *Xanthomonas* have been reported previously. The presence of contaminating DNA is a particular challenge for researchers working with samples containing a low microbial biomass. In these cases, the low amount of starting material may be effectively swamped by the contaminating DNA and generate misleading results (Salter et al., 2014).

Second, DNA library preparation, based on fragment amplification through PCR with barcoded primers, is another step in which it is possible to introduce additional biases. The choice of primers and targeted variable regions will bias identification and quantification (Soergel et al., 2012; Bokulich and Mills, 2013). Additionally, in any PCR- and primer-based taxonomic investigation, members of a microbial community may be omitted, distorted, and/or misrepresented, typically due to primer mismatches or PCR biases (Acinas et al., 2005; Hong et al., 2009; Lee et al., 2012; Pinto and Raskin, 2012; Logares et al., 2014). On the contrary, primers might show variability in their amplification efficiency by for example, favoring certain species amplification (Baker et al., 2003; Sipos et al., 2007; Klindworth et al., 2013). This preferential amplification is thought to be derived from different sources such as primer mismatches, the annealing temperature and PCR cycle numbers (Sipos et al., 2007). For instance, Sipos et al. (2007)’ studies evidenced that *A. hydrophila* and *P. fluorescens* were preferentially amplified over both *Bacillus* strains when the 63F primer was used (which contained three mismatches against DNA isolated from the *Bacillus* strains), while the 27F primer amplified all templates without bias. Interestingly, the bias introduced by primer mismatches was reduced at lower annealing temperatures.

Multiple primer pairs are available for marker genes, and each pair is associated with its own taxon biases. Marker gene databases are frequently updated, and the updated information can include new microbial lineages with suboptimal or poor binding to existing PCR primers; to maximize taxonomic sensitivity in light of these new data, primers may need to be periodically redesigned. A recent example in the literature is the modification of the most common 16S primers used 515f and 806r to remove know biases against *Crenarchaeota*/*Thaumarchaeota* and the marine and freshwater Alphaproteobacterial clade SAR11 (Apprill et al., 2015; Parada et al., 2016).

Klindworth et al. (2013) evaluated the coverage and phylum spectrum for bacteria and archaea of 175 primers and 512 primer pairs *in silico* for three amplicon size classes (100–400, 400–1000, >1000 bp), demonstrating the differences in coverage and specificity among the studied primers. Besides, this information represents a valuable guideline for selecting primer pairs that could minimize the bias in PCR-based microbial diversity studies. In the same way, probeBase^1^ is an additional online resource, providing the opportunity to evaluate the *in silico* hybridization performance of oligonucleotides, as well as finding suitable hierarchical probes that could target an organism or taxon of interest at different taxonomic levels (Greuter et al., 2016).

The ideal marker gene should have conserved regions that flank variable regions. The conserved regions allow primer design to amplify multiple taxons at ones. Ribosomal rRNA genes fit this description and have been widely used for identification of bacteria/archaea (16S) and fungi (ITS) (Gilbert et al., 2010). However, ribosomal RNA genes show copy-number variation, with very disparate number of copies per taxa (from one in many species to up to 15 in some bacteria and to hundreds in some microbial eukaryotes) biasing conclusions related to the abundance of the organisms.

To evaluate the entire microbial community in the specific case of the wine ecosystem, it is necessary to strike an appropriate balance between amplifying all members of every taxon (high coverage) and obtaining the highest taxonomic resolution possible, e.g., to be able to discriminate among closely related species (**Figure 1**). Each marker shows differences in its discrimination power at intra-genera as well as at intra-species level. Thus researchers must have that in mind when designing their project, in order to choose the most appropriate molecular marker to answer their particular question/s. For instance, primer pair 515f/806r is the most widely used for targeting the V4 region of for bacteria/archaea (Parada et al., 2016), and this combined with Illumina sequencing has been used to characterize the microbiomes of numerous environments (Caporaso et al., 2012), vine and wine environments among them. Data from high diverse environments, as Sakinaw Lake, showed species resolution level from 49.4% of the 16S V4 sequences classified compare with 74.5% using full 16S. Although the relative classification differences at the sequence level do not directly translate to differences in community representation (Singer et al., 2016). However, vine and wine samples have the added difficulty in that mitochondrial and chloroplast DNA can be amplified with these V4 region primers and thus grapevine plastid sequences overwhelm the sequencing. Researcher have two ways to avoid this problem: design primers that mismatch mitochondrial/chloroplast sequences or add blocking reagents that bind these sequences (Lundberg et al., 2013). Besides, the V4 domain of the 16s rDNA gene is considered to be the most suitable marker for capturing the bacterial community in wine, as it is able to reliably discriminate LAB to genus-level (Bokulich et al., 2012). However, in fermentative systems, some species of LAB are considered wine spoilers while others exhibit malolactic activity, thus it might be essential to reach to species level (Bokulich and Mills, 2012) and/or strain level, in order to have a more comprehensive view of the community. Unfortunately, currently available amplicon sequencing markers are unable to capture that level of resolution in all taxa. These limitations could be overcome by combining several techniques such as genera specific T-RFLP or qPCR and amplicon sequencing.

Third, important sources of artifacts are also derived from the High-throughput sequencing technology chosen. While pyrosequencing introduces homopolymer errors (indel error), Illumina sequencing has average substitution errors at 0,0086 sequencing rate (Schirmer et al., 2015). Sequencing platforms also show a disparity in sequencing depth (number of reads per run) and read length. Illumina MiSeq is the most commonly used sequencer for amplicon sequencing due to its high coverage with a total nucleotide sequenced of 15GB allowing sequencing the abundant and rare community giving a deep view of the community composition. However, llumina sequencing is characterized by a short variable region sequencing (2 × 300 bp vs. 700 bp in 454). Currently, nearly full-length rRNA gene sequencing is possible with PacBio and Nanopore technologies (Benitez-Paez et al., 2016; Schloss et al., 2016).

Finally, one of the biggest limitation of amplicon sequencing techniques relays on its inability to address a functional characterization of the microbial communities. There are many desired microbial functions in winemaking, mainly related to alcoholic and malolactic fermentations, and diversity of genes related to those functions may influence winemaking more than just taxonomic diversity. In addition, closely related strains with highly similar 16S rRNA gene or ITS sequences contain different fermentation-related genes (Knight et al., 2015) and thus that strain diversity remains hidden in current amplicon sequencing studies. Single-cell genomics emerges as a potential strategy that could help to obtain a deeper knowledge into species-strain level diversity. This strategy is powerful when the targeted organism is dominant or high abundant in a low species richness ecosystem. However, in highly diverse ecosystems or when the species to be targeted is low abundance, it may require a higher sorting throughput, specific labeling with fluorescent probes or a previous cultivation step, all of which could contribute to biases.

Alternatively, shotgun metagenomic sequencing would also reveal functional genes in addition to rRNA genes, allowing a more comprehensive genomic and functional representation through whole-genome sequencing (WGS) of complete communities, but the cost and the number of reads needed to estimate the environmental population is high compared to PCR-based approaches. Even more in wine samples, as a very deep sequencing is required to detect microbes due to an overabundance of plant DNA (Zarraonaindia et al., 2015), making this method costly for a large number of samples.

Metatranscriptomics is emerging as a powerful technology for the functional characterization of microbial communities that can reveal both the taxonomic composition and active biochemical functions of the detected organisms. These approach is of especial interest in wine environment, as amplicon sequencing is not able to discriminate among living or dead organisms, nor the metabolically active or inactive organisms. However, the high sequencing depth needed and the high cost associated with the sequencing of each sample limits the number of samples that could be surveyed within a project currently. In addition, challenges associated with this technique include among others, the lack of established reference genomes to annotate the short reads generated in the sequencing and the high computational effort needed for the analyzes. Being a technique still in its infancy, new analysis tools and standardized pipelines are under development. In this context, the next section aims to summarize critical concepts and sources of biases in NGS analysis.

## Bioinformatics and Predictive Methods to Uncover the Microbial *Terroir*

Along with the relative ease with which thousands of organisms can be detected in samples via 16S/ITS sequencing, a whole host of bioinformatics approaches have been developed to extract meaningful results from the large datasets that are generated. The bioinformatics challenge comes in at least two parts (I) preprocessing the datasets into a collection of representative reads (or operational taxonomic unit – OTU) that can be associated with databases of known species and (II) associating the collection of species inferred in a sample (known and newly detected) with properties of the sample in order to study the relationships between the microbiome and the *terroir*.

In the first stage, the large amounts of raw sequencing reads are processed (trimming adaptor sequences, merging forward and reverse sequences, filtering on read quality) before finally being dereplicated into a collection of unique sequences. There is a lot of software available to perform these tasks and they often are part of packages that offer an entire processing pipeline (USEARCH, vsearch, FASTX-Toolkit) (Edgar, 2013; Rognes et al., 2016). The unique sequences are then clustered according to sequence similarity, choosing a relatively arbitrary cutoff at 97% identity (Seguritan and Rohwer, 2001), resulting in a set of OTUs that are each assumed to be originating from a specific organism. In other words, OTUs are proxies for microbial species in the sample (Schloss et al., 2009; Caporaso et al., 2010).

Although conceptually simple, this step poses major challenges both computationally and in terms of biases that might potentially bleed into subsequent analysis. First of all, for large sets of sequences, all against all pairwise alignments would be prohibitive, e.g., 1 million of unique sequences (commonly encountered), would require 1000 billion pairwise comparisons. This has led to comprehensive bioinformatics pipelines for OTU clustering, including the software pipelines mentioned above (USEARCH, vsearch, swarm), which all rely on clever heuristics (Edgar, 2013; Eren et al., 2013; Mahé et al., 2014, 2015; Tikhonov et al., 2015; Rognes et al., 2016) in order to accelerate this process at the expense of perfectly accurate clustering. The second challenge is to avoid biases that can occur during OTU clustering. The biases can be multifold; (a) different biological species might have the same sequence and therefore be grouped into one set, (b) sequencing errors or amplification errors (including chimeric reads) or untrimmed sequences can group sequences that have the same origin into separate groups. The first issue will underestimate biological diversity whereas the latter will overestimate it. Together these scenarios will corrupt the accurate representation of the real biological makeup of the *terroir*. This highlight again that it is important to quality trim and filter the raw sequences to minimize the risk of including artifacts in environmental data sets.

Finally, the curated OTUs are subjected to phylogenetic assignment, which aims to identify what species or genus an OTU most likely belongs to. This is achieved by comparing them with taxonomically classified sequences at databases, such as GreenGenes (for bacteria community characterization), SILVA (bacteria and eucaryotes) and Unite (for Fungi) among others. Again, a range of software is available (Qiime, UTAX, SINTAX, stampa) (Caporaso et al., 2010; Edgar, 2013, 2016). This stage is again a source of biases, partly because OTUs can represent multiple species, there is ambiguous assignment, and because too small differences that do exist could be ignored by these methods. For instance, in the case of Oligotyping, a single base pair can differentiate ecological strains (Eren et al., 2013). Furthermore, and more generally, reference databases are themselves based largely on predicted species rather than experimentally cultivated species and can thus bias taxonomic assignment. Additionally, different reference databases would yield different taxonomic assignments as a function of completeness and quality of the database (McDonald et al., 2012). Notably, if a given species is not represented within the database, sequences derived from that species would receive an incorrect assignment or remain unclassified. This is aggravated for wine and soil associated microbial sequences field, where reference databases lag behind human-associated microbes. Increasing and curating robust databases is a key goal for the scientific community (**Figure 1**). There are also other methods allowing comparison of amplicons derived from functional genes in which we might not know percent identities that correspond to taxonomic levels, but in some cases, are optimal to reflect geographical (and thus, environmental) distance (Haggerty and Dinsdale, 2017). In relation to wine related samples, cluster free methods show the potential to define the microbial *terroir* at the strain or sub-OTU level (Tikhonov et al., 2015; Eren et al., 2016).

Equipped with a dataset of biological entities in the terroir (genus- or species-level), the second bioinformatics challenge concerns associating the microbiome to the properties of the *terroir*. Depending on the aim, this can be more or less difficult. One goal is to use microbial community data to classify soil samples into types and geographical regions and, therefore, define the microbial *terroir*. Recently, Bokulich et al. (2016) demonstrated the power of this approach for classifying Californian regions and fermentation metabolites based on microbial abundances in musts. However, if species or even strain information is required to establish an association between microbiome and specific wine making properties, then the taxonomic assignment is essential and can make or break an analysis depending on the resolution it achieves and the biases it can prevent.

Apart from the nature of the question, generally, the structure of OTU abundance data poses some challenges that need to be carefully taken into account. Because the species can occur in very different abundances (often spanning several orders of magnitude), the collection of species across samples can greatly vary. This leads to a very sparse dataset, which is defined as a dataset with many zero values. These zero values can be problematic as they could entertain multiple hypotheses; for instance, a zero count in a sample could be because a species is not present, or because it just has not been detected. This can lead to biased comparisons between samples. One way to deal with this is to use distance metrics that do not consider these situation (e.g., Bray-Curtis) or that specifically include a phylogenetic tree that allows to relate species information into meaningful groups. Preprocessing of OTU data from raw counts to a value that makes samples comparable to each other is the next step. This is also referred to as normalization and there are number of analytical choices available (Segata et al., 2011; Paulson et al., 2013) depending on whether low-abundance species or high abundance species should have more of an impact in the analysis inquestion. For instance, counts can be converted to frequencies (divide the number of reads by the total number of reads in the sample). The performance of these techniques given OTU table peculiarities has been tested elsewhere (McMurdie and Holmes, 2014; Weiss et al., 2016). This is also a crucial step when applying machine learning techniques.

With a preprocessed dataset available, the probe community level differences between samples, can be studied with supervised and unsupervised machine learning techniques. Unsupervised learning categorizes samples based on OTU abundances without prior knowledge of the sample phenotypes. Principle component analysis (PCA or more commonly PCoA) and clustering algorithms can be used to gain a high level view over differences in samples. These analyses are largely exploratory and provide visual evidence of community differences. If information regarding the *terroir* is available, or there are some clearly defined groups that are to studied, supervised learning techniques can be applied to for instance classify new samples based on past community characterizations (Bokulich et al., 2016). Distinct wine regions, types and tastes make wine related samples well-suited for these classification methods (Statnikov et al., 2013). Different software packages are available to perform these methods and can be more or less adapted to the study of metagenimics problems (vegan, phyloseq, Qiime, mothur).

Another method to extract knowledge from microbiome data is to consider it as a network of interactions between individual strains. Aside from the impact of single strains in plant health (pathogens, symbionts) and wine characteristics, or spoilage potential, these strains impact wine production not in isolation but instead as members of complex microbial communities. Much research now focuses on these community level effects that can impact plant phenotypes such as flowering time (Wagner et al., 2014).

These predictive technologies allow to make initial inferences about whether these differentially abundant single OTUs cause certain phenotypes. However, they will requires further testing, likely with pure culture treatments. One excellent example of going from correlation to causation is the use of pure fungal and oomycete cultures in a common garden to confirm single strain effects on the overall microbial community structure associated with *Arabidopsis thaliana* (Agler et al., 2016).

Defining the microbial *terroir* with bioinformatics is only an early step to understand how microbes shape each step in winemaking. Wine imparts its taste and smell via metabolites, many derived from the grapes and many derived from or modified by microbes. Identifying which microbes influence these processes is key to defining how they affect the sensory profile of wines. As we add genomic sequences to our reference database we will be able to leverage annotated sequences to predict metabolic capacity for each microbe. Genome scale metabolic models (GSMM) combined with flux balance analysis allows for analysis of metabolic outputs given a set of inputs (Varma and Palsson, 1994). Furthermore, GSMMs can expand to community-level models (Zomorrodi and Maranas, 2012; Khandelwal et al., 2013; Louca and Doebeli, 2015) to uncover how microbes synergistically create complex wine metabolite profiles. Going forward, it will be critical not only to define which microbes created your favorite wine but also how their metabolisms shaped the taste of that wine. Thus, viticulture will benefit very much from the generation of commercial platforms that enable studying vine and wine microbiome and wine metabolome. Currently such platforms are already in place, with WineSeq^®^ – (Biome Makers, Inc.)^2^ allowing wine microbiome characterization through NGS and Wine Screener^®^ – (Bruker)^3^ allowing wine metabolome analysis by nuclear magnetic resonance. These tools are based on robust databases and allow both producers and regulatory councils from appellations of origins to establish ‘standard profiles’ for their wines, and better understand the microbial and chemical bases of their distinctive *terroir*.

## Conclusion

In this article, the impact of NGS technologies in vine and wine microbiology has been reviewed. Regarding the importance of microbiome in viticulture and enology, the role of microorganisms in the chemical and nutritional properties of vineyard soils, crop health and yield, and also in the later fermentation performance and wine flavor are the main challenges to explore using –omics tools. For that purpose, certain technical aspects should be improved at laboratory stages, such as universal DNA&RNA extraction protocols to avoid biases, and improved sequencing approaches to increase microbiome resolution and quantification. It is also important to develop robust and curated databases to improve taxonomic assignments (**Figure 1**). Finally, it is time to develop big data works, using statistical data-mining and machine learning tools to solve, in a holistic systems-biology view, the above-mentioned challenges in wine industry.

## Author Contributions

IB and AA conceived the work. IB, AP, and AA wrote the “Introduction” section. IB and IZ wrote “The microbiome of vine and wine: a review” section. AA, IZ, and MP wrote “NGS microbial profiling: key steps, biases and limitations” section. IZ and MP wrote “Bioinformatics and predictive methods to uncover the microbial terroir” section. Finally, IB edited the final version of the manuscript.

## Conflict of Interest Statement

The authors declare that the research was conducted in the absence of any commercial or financial relationships that could be construed as a potential conflict of interest.

## Footnotes

1 http://www.probebase.net

2 http://www.wineseq.com/

3 http://www.bruker.com/products/mr/nmr/food-screener/wine-profiling/overview.html
