# Corrigendum: From Vineyard Soil to Wine Fermentation: Microbiome Approximations to Explain the “*terroir*” Concept

**DOI:** 10.3389/fmicb.2017.01065

**Published:** 2017-06-12

**Authors:** Ignacio Belda, Iratxe Zarraonaindia, Matthew Perisin, Antonio Palacios, Alberto Acedo

**Affiliations:** ^1^Biome Makers Inc.San Francisco, CA, United States; ^2^Department of Microbiology, Biology Faculty, Complutense University of MadridMadrid, Spain; ^3^Department of Genetics, Physical Anthropology and Animal Physiology, University of the Basque CountryLeioa, Spain; ^4^IKERBASQUE – Basque Foundation for ScienceBilbao, Spain; ^5^Laboratorios Excell IbericaLogroño, Spain

**Keywords:** NGS, wine microbiome, vine health, soil microbiome, metagenomic analysis, bioinformatic tools and databases, 16S rRNA gene sequencing

In the original article, there was an error in the Conflict of Interest statement. The correct version appears below. The authors apologize for this error and state that this does not change the scientific conclusions of the article in any way.

## Conflict of interestl statement

IB, MP, AP, and AA are employees or active collaborators of Biome Makers Inc., which also funded this study. AP is affiliated with Excell Iberica Laboratories. The other author declares that the research was conducted in the absence of any commercial or financial relationships that could be construed as a potential conflict of interest.

